# Impact of Curcumin on Frozen Bovine Sperm Quality and In Vitro Bovine Oocyte Maturation ^†^

**DOI:** 10.3390/vetsci12050441

**Published:** 2025-05-05

**Authors:** Hao Lin, Zhiye Hu, Yang Li, Yingchun Li, Wenao Ma, Shoujie Zheng, Jianye Zhou, Zhihui Zhao, Shangquan Gan, Zhibao Chen, Namula Zhao

**Affiliations:** 1 College of Coastal Agriculture Science, Guangdong Ocean University, Zhanjiang 524088, China; linhao4@stu.gdou.edu.cn (H.L.); huzhiye@stu.gdou.edu.cn (Z.H.); 13178572391@stu.gdou.edu.cn (Y.L.); liych@gdou.edu.cn (Y.L.); 2112204018@stu.gdou.edu.cn (W.M.); shangquangan@gdou.edu.cn (S.G.); chenzb@gdou.edu.cn (Z.C.); 2 Animal Husbandry Technology Promotion Station, Zhanjiang Municipal Bureau of Agriculture and Rural Affairs, Zhanjiang 524088, China

**Keywords:** bovine, sperm, frozen, IVM

## Abstract

This study aimed to analyze the effect of curcumin on the antioxidant properties and fertility of freeze–thawed bovine spermatozoa and bovine oocytes. In this study, curcumin concentrations of 0, 5, 10, 25, and 50 µM were added bovine sperm cryopreservation solution and oocyte IVM medium to assess sperm quality, antioxidant properties, oocyte maturation, IVF rate, and embryonic development. The results demonstrated that adding curcumin to the cryopreservation solution significantly improved the viability, motility, and acrosome integrity of bull sperm after freezing and thawing (*p* < 0.05). The addition of 25 µM curcumin resulted in the best sperm quality. Analysis of antioxidant capacity showed that 25 µM curcumin significantly increased the activities of MMP and antioxidant enzymes, such as CAT, SOD, and GSH-PX, and lowered the levels of MDA and ROS (*p* < 0.05). Adding curcumin to the in vitro maturation medium notably enhanced the maturation rate and decreased DNA fragmented nuclei of bovine oocytes (*p* < 0.05), with optimal outcomes observed at 25 and 50 µM curcumin. Totals of 25 and 50 µM curcumin markedly elevated GSH and MMP (*p* < 0.05), reduced ROS and malondialdehyde concentrations (*p* < 0.05), and significantly enhanced fertilization rates and blastocyst formation (*p* < 0.05). In conclusion, incorporating curcumin into both the bovine semen cryopreservation solution and the oocyte IVM medium significantly improved the quality of frozen–thawed sperm, antioxidant activity, oocyte maturation, IVF rate, and embryonic development.

## Simple Summary

Bovine sperm cryopreservation and oocyte maturation in vitro were found to be susceptible to oxidative stress damage. A plant polyphenol called curcumin can be extracted from the rhizomes of curries, tulips, turmeric, and other plants. Curcumin has beneficial biological effects, such as antimicrobial, antioxidant, and anticancer actions. It can also inhibit the production of reactive oxygen species (ROS), scavenge free radicals, enhance antioxidant enzyme activity, and effectively combat oxidative stress. In this study, we investigated the effects of curcumin on oxidative stress and embryo development in bovine sperm cryopreservation and oocyte in vitro maturation by adding different concentrations of curcumin to bovine sperm cryodilution and oocyte in vitro maturation medium. The results showed that curcumin effectively alleviated the oxidative stress damage in bovine sperm cryopreservation and oocyte in vitro maturation, improved sperm quality, oocyte maturation rate, fertilization rate and blastocyst rate, and promoted embryo development.

## 1. Introduction

The expansion of optimal breeding stocks of animals through in vitro embryo production technology is a current trend of biotechnology development, since the production of livestock products is insufficient to meet the needs of the growing human population. In vitro embryo production can shorten the animal generation interval, eliminate geographical restrictions and individual differences in animals, accelerate genetic improvement and breeding, save endangered animals, improve the reproductive efficiency of high-quality populations, and provide an important reproductive breeding method in intensive and large-scale animal breeding programs [1]. In vitro oocyte maturation (IVM), in vitro fertilization (IVF) of mature oocytes, and in vitro culture of embryos are three integrated technologies used for in vitro embryo production. However, in vitro embryo generation techniques remain incomplete, with shortcomings such as poor embryo developmental potential and low survival rate. Numerous investigations have demonstrated that the primary causes of these shortcomings are the reduced quality of IVM oocytes and the decreased fertilization rate of cryopreserved sperm. Semen cryopreservation is one of the most important ways to preserve animal genetic breeding resources. Maintaining sperm viability is crucial for successful IVF and subsequent embryo maturation.

Oxidative stress is one of the biggest challenges facing sperm cryopreservation and oocyte maturation in vitro. Excess reactive oxygen species (ROS), such as the free radical superoxide anion (O^2−^), hydroxyl radical (-OH), and non-free radical hydrogen peroxide (H_2_O_2_), are the primary cause of oxidative stress [2]. Naturally occurring byproducts of regular oxygen metabolism, ROS are crucial for cell signaling. A moderate amount of ROS can promote intracellular signaling and regulation, whereas excessive cellular accumulation of ROS causes a state of oxidative stress, where unsaturated phospholipids are destroyed and malondialdehyde (MDA) levels are elevated, thereby damaging cells [3].

The resilience of spermatozoa during cryopreservation is primarily influenced by the membrane lipid profile, which is low in cytoplasmic antioxidant enzymes and rich in unsaturated fatty acids, making the membrane lipids more sensitive to ROS, which reduces the fluidity and changes the permeability of membranes. Sperm experience cold shock when the outside temperature decreases from room temperature to −20 °C or −196 °C, and reduced temperatures also result in the production of free radicals and ROS [4]. Excessive free radicals and ROS can induce sperm lipid peroxidation, resulting in sperm lipid damage, mitochondrial dysfunction, DNA methylation, and, in severe cases, sperm death. It has been found that the main pathway by which ROS affect sperm viability is the inhibition of enzyme activity in processes such as oxidative phosphorylation and glycolysis in the inner mitochondrial membrane: inhibition which prevents the synthesis of ATP and lowers sperm viability [5,6].

The primary factor behind the inefficiency of both IVM and in vitro embryo development is oxidative stress. During oocyte maturation in vitro, disruptions in the surrounding environment, the content of culture medium, oxygen levels, and other variables result in the generation of ROS. Oocyte cultivation in vitro lacks the protection of non-enzymatic antioxidant enzymes compared with in vivo maturation, and the oxygen-rich conditions in vitro elevate the production of free radicals, triggering oxidative stress. This surge of ROS and the buildup of free radicals results in the oxidization of amino acids [7] and disruption of the oocyte plasma membrane and nucleic acid structure [8], leading to oocyte meiotic arrest and mitochondrial dysfunction [9], DNA damage and apoptosis, and diminished blastocyst formation, impacting embryo growth and viability [10]. The addition of antioxidants to semen cryopreservation solution and oocyte maturation medium provides an effective method to reduce oxidative damage to semen and oocytes in vitro.

Because of their low toxicity, plant extracts have been employed as natural antioxidants in bovine semen cryopreservation and oocyte culture in vitro [11], such as resveratrol [12], epigallocatechin gallate [13], and ferulic acid [14]. Research indicates that resveratrol enhances antioxidant activity and sperm fertilization potential during bovine semen cryopreservation, and reduces ROS production, DNA fragmentation, and lipid peroxidation [15]. The inclusion of 1 µM resveratrol in maturation medium enhanced bovine oocyte cleavage and blastocyst hatching rates [16]. Wang et al. [17] demonstrated that ferulic acid reduced oxidative stress in bovine oocytes during IVM, thereby improving the quality of both oocytes and early embryos.

Curcumin is a plant-based polyphenol mainly extracted from turmeric rhizomes [18]. Curcumin has been employed extensively in food, healthcare, cosmetics, and other industries because of its biological properties, which include anti-inflammatory, antioxidant, antibacterial, and anticancer properties [19,20]. Natural curcumin has an antioxidant activity 8-times more than that of vitamin E and 2.75-times greater than that of vitamin C. The antioxidant capacity of curcumin mainly comes from its chemical structure, which includes a number of functional groups, such as the β-diketone group, carbon-carbon double bond, benzene ring, and also phenolic hydroxyl group and methoxy substituents, the primary source of its antioxidant properties. Curcumin can effectively transfer electrons or provide hydrogen atoms, thereby scavenging reactive free radicals in oxidative reactions, activating cytoprotective signals, and mitigating oxidative damage to cells. Curcumin has a dual ability to regulate antioxidant activity, by maintaining the dynamic balance of body ROS content by scavenging hydrogen peroxide and nitric oxide free radicals, while also stimulating the action of antioxidant enzymes such as glutathione peroxidase (GSH-PX), catalase (CAT), and total superoxide dismutase (T-SOD). According to Namula et al. [21], adding 10 µM curcumin to culture media during pig oocyte IVM enhanced the maturation and fertilization rates of oocytes, and protected the oocytes from oxidative stress caused by H_2_O_2_. However, the antioxidant role of curcumin during the IVM of bovine oocytes and the subsequent development of embryos has yet to be thoroughly examined.

In this study, different concentrations of curcumin (0 to 50 µM) were added to bovine sperm cryopreservation solution and oocyte IVM medium. Semen quality was assessed by evaluating sperm viability, vigor, plasma membrane integrity, and acrosome integrity following freezing and thawing. Furthermore, antioxidant parameters, such as ROS, CAT, SOD, GSH-PX, MDA, and MMP, were measured, along with IVF parameters, including fertilization, oocyte cleavage, and blastocyst formation rates. This study aimed to analyze the impact of curcumin on frozen thawed sperm quality and antioxidant properties, as well as explore the underlying mechanisms by which curcumin influences the IVM of bovine oocytes, with the objective of enhancing livestock development and improving bovine sperm quality.

## 2. Materials and Methods

### 2.1. Chemicals

Unless otherwise specified, curcumin and other chemicals were purchased from Macklin Biochemicals (Shanghai, China). Curcumin (3.68 mg) was dissolved in DMSO (1 mL) to obtain a 10 mM curcumin base stock solution.

### 2.2. Experiment 1

#### 2.2.1. Semen Collection and Cryopreservation

Bulls were provided by Zhanjiang Agricultural Machinery Technology Promotion Station, Zhanjiang City, Guangdong Province. A selection of 6 healthy and robust bulls, aged 4 to 6 years, exhibiting optimal body conformation and strong reproductive function, were selected. Semen samples were collected from 6 bulls using the pseudo-vaginal method [22] and stored in a 37 °C Thermos and delivered to the laboratory within 1 h. A total of 30 ejaculates were collected twice a week (5 ejaculates per bull). Semen samples with ≥70% sperm viability were selected for mixing and cryopreservation.

The semen cryodilution formulation was referred to Namula et al. [23], and 0, 10, 20, 50, and 100 µM of curcumin was added to the first solution. Semen samples were diluted to 4 × 10^8^ sperm/mL. The composition of the first solution consisted of 2.9 g Tris-base, 0.4 g fructose, 1.59 g citric acid, 20 mL of egg yolk, and 200 µL of amikacin sulfate at 0.2 mg/mL, made up to 100 mL using distilled water. Centrifuge tubes (15 mL) with semen samples were submerged in 300–350 mL of purified water at 25 °C in a glass beaker and then equilibrated at 4 °C for 2.5 h. The same volume of the second dilution was added to the tube [93.2% (*v*/*v*) first dilution, 6% (*v*/*v*) glycerol, 0.8% (*v*/*v*) EQUEX STM (Miyazaki Kagaku, Tokyo, Japan)] to achieve final concentrations of 0, 5, 10, 25, and 50 µM curcumin and incubated at 4° for 10 min. The final sperm concentration of 2 × 10^8^ sperm/mL, and 3% (*v*/*v*) glycerol. The diluted semen was added to 0.25 mL frozen straws and sealed with sealing powder. The chilled tubes were then set on Styrofoam plates, exposed to liquid nitrogen at −196 °C for 10 min, and subsequently stored in liquid nitrogen tanks.

#### 2.2.2. Sperm Viability and Vigor Assay

The chilled cattle semen was swiftly warmed in a 37 °C water bath for 10 s. A 10 µL semen sample was then transferred to a warmed slide at 37 °C and sealed with a coverslip. Spermatozoa viability was assessed under a microscope (200× magnification), with at least three or more different fields of view observed for each sample. Viability was assessed via optical microscopy, by assigning different indices (100%, 75%, 50%, 25%, 0%) to different motility types of spermatozoa, with sperm viability calculated as shown in the formula [24]:

Sperm viability = (A × 100% + B × 75% + C × 50% + D × 25% + E × 0%)/100

where A is the number of spermatozoa moving forward in a straight line in a vigorous manner, B is the number of spermatozoa moving forward in a slow manner, C is the number of spermatozoa moving in a circle and rotating in place, D is the number of spermatozoa doing a pendulum motion relying on the tail rotating in place, and E is the number of non-motile or dead spermatozoa. Four replicate tests were performed.

#### 2.2.3. Assessment of Sperm Plasma Membrane Integrity

The hypotonic swelling method was used to evaluate the integrity of the sperm plasma membrane [25]. A 10 µM semen aliquot was mixed with 90 µL hypotonic solution, prepared with 9 mg/mL fructose and 4.9 mg/mL sodium citrate dihydrate. The sample was then placed in a water bath at 37 °C for 30 min. After incubation, 10 µL of the solution was mixed with eosin staining solution (1:1 in volume) and used to coat a slide. After natural air-drying, spermatozoa were observed under a microscope (200× magnification), and the tail curling rate was recorded. More than 200 spermatozoa were observed for each sample. A total of four replications were conducted.

#### 2.2.4. Acrosome Integrity Assay

The functionality of the sperm acrosome was determined by the Cauloblue G250 staining method [25]. To begin, 100 µL of semen was centrifuged at 2000 rpm for 3 min, the resultant supernatant was discarded, and sperm were rinsed twice with 100 µL of DPBS. After washing, spermatozoa were combined with 1 mL of 4% paraformaldehyde and incubated for 10 min, then the samples were centrifuged at 2000 rpm for 10 min. The supernatant was removed, and sperm were washed with 1 mL DPBS, then resuspended with 20 µL DPBS, 10 µL of which was taken for coating a slide, which was soaked in Caulmers Brilliant Blue staining solution for 30 min, rinsed, and then dried naturally. Stained sperm acrosomes were visualized under a microscope (400× magnification), with more than 200 spermatozoa observed per sample. If the acrosome area was blue and unbroken, the acrosome was complete, and if the acrosome area was lightly colored or broken, the acrosome was incomplete. The test was repeated four times.

#### 2.2.5. Antioxidant Property Assay

CAT, SOD, GSH-PX, MDA, and MMP kits were provided by Elabscience Biotechnology Co. (Wuhan, China). The ROS kit was provided by Beyotime (Shanghai, China). For kit use, refer to Lin et al. [26].

CAT enzyme assay: The CAT enzyme content was determined according to the instructions of a CAT test kit. The frozen tube was warmed in a 37 °C water bath, then washed twice with DPBS, mixed with reagent I and incubated at 37 °C for 5 min, mixed with reagent II and incubated for 1 min. Finally, reagent III and reagent IV were added, mixed and incubated at 10 min. The absorbance of the sample was determined at 405 nm by an enzyme labeling instrument.

SOD enzyme assay: Samples were processed following the SOD kit protocol and a standard curve was generated. Sperm were mixed with kit reagents and incubated at 37 °C for 20 min, and then the absorbance at 450 nm was detected by an enzyme marker.

GSH-PX enzyme assay: The GSH-PX enzyme levels in sperm were assayed following the procedures outlined in the GSH-PX assay kit, and a standard curve was plotted. Reagent I was mixed with 0.02 mL of 1 mmol/L GSH and 0.02 mL of spermatozoa in a 37 °C water bath for 5 min. Subsequently, 0.01 mL of Reagent I was added and the sample incubated at 37 °C for 5 min. Then, 0.2 mL of Reagent II was mixed and the sample centrifuged at 3100 rpm for 10 min. A 0.1 mL aliquot of the resulting supernatant was used for the color development process. Following a 5 min incubation, the absorbance at 412 nm was measured, with the experiment replicated four times.

Sperm ROS assay: Samples were processed following the ROS kit instructions. Spermatozoa were thawed and mixed with the DCFH-DA working solution, incubated at 37 °C protected from light for 30 min, mixed every 5 min, washed twice with DPBS and the precipitate resuspended. Fluorescence evaluation utilized a flow cytometer with excitation at 488 nm and emission detection at 525 nm, with four replicate measurements conducted.

MDA assay: Samples were processed according to the instructions of the MDA test kit. An aliquot of 0.25 mL was used to detect the absorbance at 532 nm by the enzyme labeling instrument. Four replicates were performed.

MMP assay: Samples were processed following the protocol of an MMP test kit. Samples were washed with DPBS once or twice and mixed with JC-1 staining solution. Then samples were incubated in the dark at 37 °C for 30 min, then rinsed with DPBS once or twice before being analyzed by a flow cytometer. The FITC/PE channel was used to obtain data, and the size of MMP was indicated by the ratio of red to green fluorescence. Four replicate experiments were performed.

#### 2.2.6. In Vitro Fertilization

In vitro fertilization methods was referred to Namula et al. [21]. Frozen bovine semen tubes were extracted from liquid nitrogen and thawed for 8 to 10 sec in a water bath set to 37 °C. Then, the mixture was centrifuged at 630× *g* for 5 min. After centrifugation, the supernatant was discarded. Semen was washed twice and the sperm concentration was adjusted to 2 million sperm/mL by adding fertilization medium (IVF100,Research Institute for the Functional Peptides Co., Yamagata, Japan).

Granulosa cells were removed by repeated pipette aspiration of the cumulus oocyte complexes (COCs) with the use of hyaluronidase, and mature COCs containing the first polar body were collected. In total, 250 μL of diluted semen was added to 250 μL of fertilization medium, which contained 50–55 mature oocytes. The fertilization rate was detected after incubation of the spermatozoa and oocytes in an incubator at 38.5 °C, 5% CO_2_, 5%O_2_, and 90% N_2_ for 18 h. Fertilized eggs were removed with a pipette and placed into an in vitro culture medium with 20–30 fertilized eggs per well. Then, 400 µL of mineral oil was applied to the samples, which were maintained at 38.5 °C, 5% CO_2_, 5%O_2_, and 90% N_2_ for 1 week. The blastocyst formation rate was assessed on the seventh day.

### 2.3. Experiment 2

#### 2.3.1. Oocyte Retrieval and IVM Culture

Ovaries from healthy cows were obtained from the abattoir and sent to the laboratory within an hour, enclosed in saline holding cups containing double antiseptic (penicillin and streptomycin sulfate) at 30 °C to 33 °C. After washing with saline containing penicillin and streptomycin sulfate at 37 °C, fluid was harvested from sinusoidal follicles measuring 3–5 mm in diameter via a single-use syringe, then centrifuged at 2000 rpm for 5 min, then the supernatant removed and transferred to a Petri dish. This sample was placed under a microscope containing a heated stage to select COCs, which were subsequently rinsed thrice in maturation solution, and then added into Petri dishes with media and curcumin dissolved in DMSO for in vitro maturation. Culture media was based on the formulation detailed by Namula et al. [27]. Culture media was contained medium 199 with Earle’s salt solution culture (Invitrogen Co., Carlsbad, CA, USA). The medium was supplemented with 0.02 AU/mL of follicle stimulating hormone (Kyoritsuseiyaku, Tokyo, Japan), 5% (*v*/*v*) fetal bovine serum (Invitrogen, USA), 0.6 mM of cysteine (Sigma-Aldrich, Shanghai, China) and 50 μg/mL gentamicin (Sigma-Aldrich, Shanghai, China). Curcumin dissolved in dimethyl sulfoxide was added for a final concentration of 0, 5, 10, 25, and 50 mM in the IVM medium. Twenty COCs were incubated in an incubator at 38.5 °C and 5% CO_2_ for 24 h, with subsequent assessment of maturation and DNA fragmented nuclei of oocytes.

Oocyte maturation rate and DNA fragmented nuclei were detected by a combined technique of nuclear staining and terminal deoxynucleotidyl transferase (TDT)-mediated dUTP Nick End Labeling (TUNEL). The staining method was based on the steps detailed based on Namula et al. [21]. Mature bovine oocytes were treated with hyaluronidase to remove granulosa cells and were washed three times with PBS-PVA. Oocytes were fixed in 4% paraformaldehyde for 15 min at room temperature and washed three times with PBS-PVA after fixation, followed by permeabilization of the oocytes in DPBS containing 0.1% (*v*/*v*) Triton-X100 for 15 min. after PBS-PVA washing, the oocytes were added to DPBS containing 10 mg/mL bovine serum albumin (Sigma-Aldrich, Shanghai, China). Oocytes were incubated overnight at 4 °C. After incubation, PBS-PVA washed, and TUNEL staining was performed by adding fluorescein-conjugated dUTP and TdT (TUNEL reagent; Roche Diagnostics, Tokyo, Japan) for 1 h at 38.5 °C. The oocytes were then counterstained with 1 mg/mL DAPI for 10 min, washed with PBS-PVA, and treated with anti-bleaching solution (Molecular Probes, Eugene, OR, USA) before placing the oocytes on slides, covering the coverslips and sealing with clear nail polish. Oocytes were observed using a fluorescence microscope. Oocytes were determined to be in the germinal vesicle (GV), middle II (MII) stage based on chromatin structure and were counted. TUNEL-labeled nuclei were counted to assess DNA fragmented nuclei in oocytes after mature culture.

#### 2.3.2. Oocyte ROS and GSH Assay

Following the instructions of the kit (Beyotime, Shanghai, China), COCs were treated with hyaluronidase to isolate the oocytes. The oocytes were then flushed with PBS-PVA three times, then carefully transferred to treated Petri dishes containing IVM medium supplemented with either 10 µM fluorescent dye H2DCFDA (Thermo Fisher Scientific, Waltham, MA, USA) or 10 µM CMF2HC (Thermo Fisher Scientific, USA). The dishes were then incubated for either 15 or 30 min. After incubation, oocytes were rinsed with PBS-PVA and positioned in 10 µL microdrops. Fluorescence examination took place under a microscope equipped with a UV filter to gauge the fluorescence intensity, with green and blue indicating ROS and GSH, respectively. Four replicate experiments were performed.

#### 2.3.3. Oocyte MMP and MDA Assay

Same as step 2.2.5.

#### 2.3.4. Assessment of In Vitro Fertilization

Same as step 2.2.6. Selection of frozen bovine semen without any drug treatment for artificial insemination.

Fertilization rate was assessed by acetic acid staining. After 10 h following fertilization of COCs, fertilized eggs were taken on slides and fixed by adding acetic acid: ethanol (1:3 *v*/*v*), fixed for 48 h and then stained with acetic acid spermatozoa (1% acetic acid spermatozoa, 45% acetic acid), and examined microscopically. Oocytes containing both female and male prokaryotes were considered fertilized and fertilization rates were counted.

Cleavage rate was assessed on day three and the blastocyst rate on the seventh day. Total cell number was counted by DAPI staining technique.

### 2.4. Statistics and Analysis

Data were graphically analyzed using Excel 2019. One-way analysis of variance was conducted on the data using SPSS 26.0 and multiple comparisons executed for each group employing Tukey’s method to detect the significance of the results. Data are presented as mean ± standard error of the mean. Different letters denote significant changes (*p* < 0.05), while identical letters signify no significant differences (*p* > 0.05).

## 3. Results

### 3.1. Impact of Curcumin on Frozen–Thawed Bovine Sperm Quality

**Figure 1:**
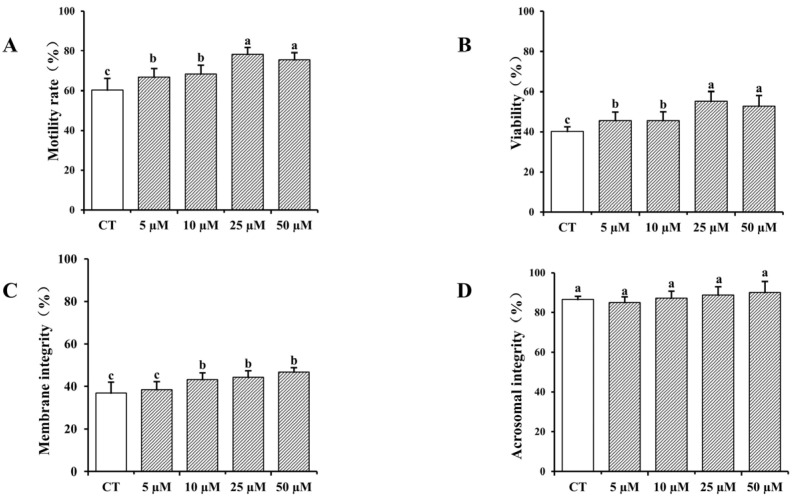
Impact of various concentrations of curcumin (ranging from 0 to 50 µM) on the viability, motility, plasma membrane, and acrosome integrity of chilled bovine sperm following freezing and thawing. (**A**) Frozen–thawed sperm motility. (**B**) Viability of frozen–thawed sperm. (**C**) Plasma membrane integrity of sperm after freeze–thawing. (**D**) Acrosome integrity of frozen–thawed sperm. A total of four replicates were performed. Identical letter notation signifies no significant difference (*p* > 0.05) and different letter notation denotes a significant difference (*p* < 0.05).

The impact of varying concentrations of curcumin on frozen–thawed bovine sperm quality is presented in Figure 1. The results indicate that the addition of curcumin to the cryopreservation solution enhanced sperm quality in a concentration-dependent manner, with higher concentrations resulting in increased sperm quality. The inclusion of curcumin in sperm cryopreservation solution markedly enhanced the viability, motility, and plasma membrane integrity of frozen–thawed spermatozoa relative to the control group (*p* < 0.05), with the most pronounced improvement with 25 µM curcumin. However, no significant differences were noted regarding acrosome integrity (*p* > 0.05).

### 3.2. Effect of Curcumin on Antioxidant Properties of Frozen–Thawed Bovine Sperm

**Figure 2:**
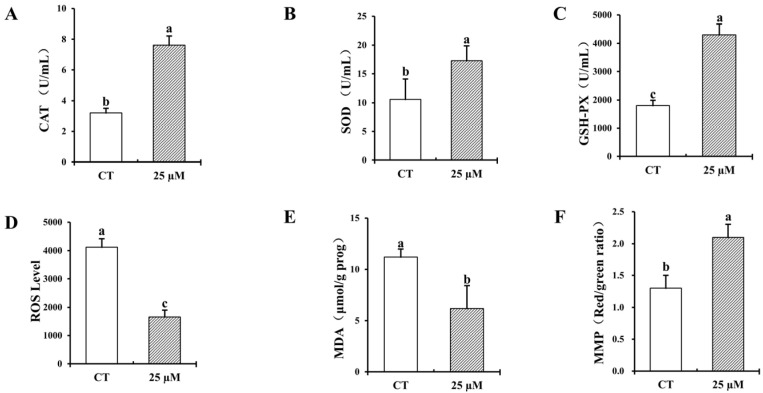
Effect of 25 µM curcumin on the activities of catalase (CAT), superoxide dismutase (SOD), glutathione peroxidase (GSH-PX), and malondialdehyde (MDA) enzymes in frozen–thawed bovine sperm. (**A**) CAT activity in frozen–thawed spermatozoa. (**B**) SOD activity in frozen–thawed spermatozoa. (**C**) GSH-PX activity in frozen–thawed spermatozoa. (**D**) Reactive oxygen species (ROS) levels in frozen–thawed spermatozoa. (**E**) MDA concentration in frozen–thawed spermatozoa. (**F**) Mitochondrial membrane potential (MMP) in frozen–thawed spermatozoa. A total of four replicates were performed. Identical letter notation signifies no significant difference (*p* > 0.05). Different letter notation denotes a significant difference (*p* < 0.05).

Figure 2 presents the effect of adding curcumin to bovine cryopreservation solution on the antioxidant properties of frozen–thawed bovine sperm. The introduction of 25 µM curcumin into cryopreserved semen markedly enhanced the activities ofantioxidant enzymes, including CAT, SOD, and GSH-PX, and significantly reduced the content of ROS and MDA, while enhancing the mitochondrial membrane potential of frozen–thawed bovine spermatozoa (*p* < 0.05).

### 3.3. Effect of Curcumin on Fertilization and Blastocyst Rates with Frozen–Thawed Bovine Sperm

**Figure 3:**
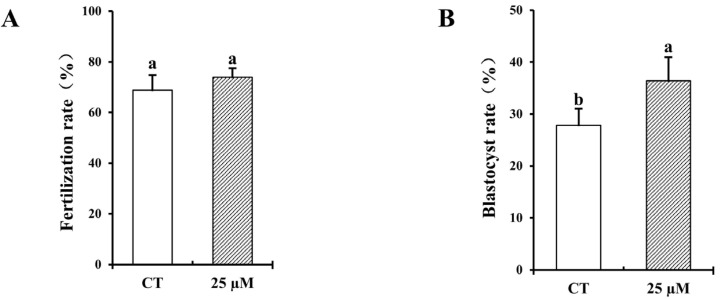
Effect of 25 µM curcumin on the fertilization and blastocyst rates with frozen–thawed bovine sperm. (**A**) Fertilization rate from frozen–thawed sperm with 25 µm curcumin. (**B**) Blastocyst rate from frozen–thawed sperm with 25 µm curcumin. A total of four replicates were performed. Identical letter notation signifies no significant difference (*p* > 0.05). Different letter notation denotes a significant difference (*p* < 0.05).

The success rates of fertilization and blastocyst formation are crucial for gauging the viability of frozen–thawed sperm in IVF procedures. As shown in Figure 3, the group treated with 25 µM curcumin demonstrated no significant differences in fertilization compared with the untreated control group (*p* > 0.05), and the 25 µM group had a significantly increased blastocyst rate (*p* < 0.05).

### 3.4. Impact of Curcumin on Bovine Oocyte Maturation and DNA Fragmented Nuclei During In Vitro Culture

Different concentrations of curcumin within the oocyte maturation medium were used to examine the impact of curcumin supplementation on bovine oocyte developmental potential during IVM. Table 1 demonstrates that incorporating curcumin into the IVM medium markedly enhanced the maturation rate of bovine oocytes at the metaphase II (MII) stage, and reduced DNA fragmented nuclei, compared with the untreated control group (*p* < 0.05). Specifically, the group treated with 25 and 50 µM curcumin markedly elevated the MII stage maturation rate over the other groups (*p* < 0.05), with no significant differences between these two concentrations (*p* > 0.05).

### 3.5. Impact of Curcumin on the Antioxidative Characteristics of In Vitro-Matured Bovine Oocytes

**Figure 4:**
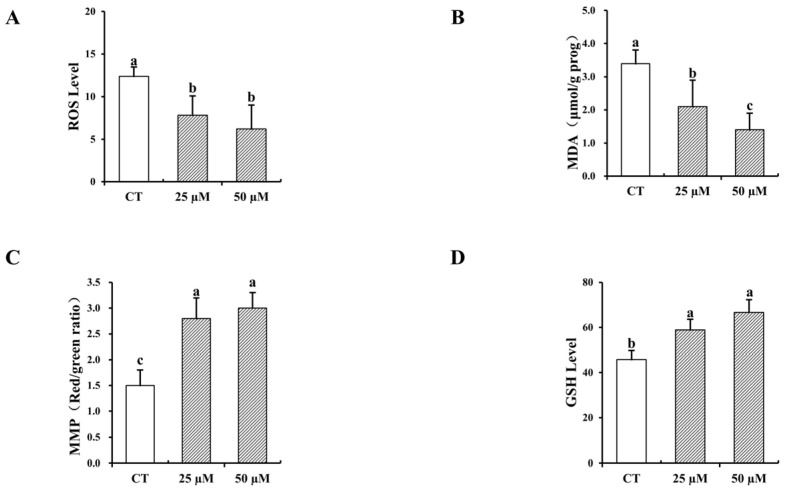
Effect of the addition of 25 and 50 µM curcumin on the levels of reactive oxygen species (ROS), malondialdehyde (MDA), mitochondrial membrane potential (MMP) and glutathione (GSH) in vitro mature oocytes. (**A**) ROS levels in the in vitro-matured oocytes. (**B**) MDA levels in the in vitro-matured oocytes. (**C**) MMP levels in the in vitro-matured oocytes. (**D**) GSH content of the in vitro-matured oocytes. A total of four replicate experiments were performed and analyzed for significance. Identical letter notation signifies no significant difference (*p* > 0.05). Different letter notation denotes a significant difference (*p* < 0.05).

As shown in Figure 4, the addition of 25 and 50 µM curcumin to IVM medium significantly increased the GSH content and MMP levels of bovine oocytes and significantly reduced the content of ROS and MDA (*p* < 0.05). Notably, the MDA content of the 50 µM curcumin group was significantly reduced compared to the 25 µM curcumin group (*p* < 0.05), and other antioxidant indices were not significantly different between the two groups (*p* > 0.05).

### 3.6. Formatting of Mathematical Components

Table 2 indicates that the fertilization rate of mature oocytes with 25 and 50 µM curcumin was notably greater than that of the control group. By the seventh day, the progression of mature oocytes was evident, with blastocyst formation rates significantly increased in the curcumin groups compared with the control (*p* < 0.05), but there was no significant difference in the cleavage rate of each group among the groups (*p* > 0.05).

## 4. Discussion

Sperm kinetic parameters, including viability and vigor, are key factors that determine the ability of sperm to reach the fallopian tube for fertilization. The integrity of both the plasma membrane and acrosome is crucial for successful fertilization. During semen cryopreservation, intracellular antioxidant enzymes are unable to protect the acrosomal and caudal plasma membranes because of the high concentration of polyunsaturated fatty acids in the sperm plasma membrane. Acetylated phospholipids in sperm tend to condense at low temperatures, leading to “cold shock” and sperm inactivation. Additionally, during cooling, sperm lose water and form ice crystals both inside and outside the cells, which disrupts membrane fluidity and structure, thereby compromising plasma membrane and acrosome integrity, leading to the loss of sperm fertilization function. Chanapiwat et al. [28] discovered that incorporating 0.25 or 0.50 mmol/L curcumin into the cryopreservation solution was effective at improving the quality of frozen preserved porcine semen, consistent with the findings of this study. However, the results of the present study showed no significant improvement in sperm acrosome integrity with the addition of curcumin. In a study by Wang et al. [25]. on the addition of CGA to ram semen dilutions, there was also no significant difference in acrosome integrity between the control and experimental groups on days 0–3, but the protective effect of CGA on the acrosome was only revealed with the increase in time. Thus, the time factor might be responsible for the lack of effect of curcumin addition on acrosome integrity in this experiment. Curcumin was found to have a strong scavenging ability for free radicals, including O^2−^, OH, and NO, and to inhibit lipid peroxidation and cold shock in spermatozoa [29]. In addition, Cojkic et al. [30] found that curcumin at a concentration of 5% positively affected sperm viability parameters and significantly reduced bacteria, suggesting that curcumin improves sperm viability and ensures plasma membrane integrity by scavenging free radicals and inhibiting lipid peroxidation and bacteria.

Studies have shown that several antioxidant enzymes, such as CAT, GSH-PX, and T-SOD, are present in semen to protect spermatozoa from damage [31]. SOD catalyzes the disproportionation of O^2−^ to H_2_O_2_ and O_2_ through the disproportionation reaction, and H_2_O_2_ is converted to H_2_O and O_2_ by CAT, thus scavenging ROS and free radicals from sperm. Therefore, the content of these antioxidant enzymes serve as a crucial metric for evaluating the antioxidant capability of spermatozoa. During sperm freezing, excessive ROS overwhelm the antioxidant defenses of spermatozoa, rendering the antioxidant enzyme levels in semen insufficient to protect sperm. This leads to lipid peroxidation and mitochondrial dysfunction. The lipid peroxidation reaction causes the decomposition of unsaturated fatty acids, resulting in the production of MDA, which serves as a marker of oxidative damage in spermatozoa. MMP is a key indicator of mitochondrial function, with alterations closely reflecting mitochondrial health. This study found that curcumin effectively enhanced antioxidant enzyme activity in bovine spermatozoa, reduced MDA levels, increased MMP, and inhibited ROS production, thereby protecting mitochondria and alleviating oxidative stress damage. Nrf2 is an important transcription factor in the oxidative stress response, whereas Keap1is a protein with a relative molecular weight of 69 kD. As a sensor of oxidative stress, in the normal physiological state, Keap1 binds to and degrades Nrf2. Upon exposure to oxidative stress, the conformation of Keap1 is modified, leading to the translocation of Nrf2 into the nucleus where it associates with antioxidant response elements (ARE), inducing and activating the transcription of antioxidant target genes (HO-1, NQO1, GSH-PX, CAT, and SOD1) [32,33], increase antioxidant enzyme activity. Curcumin possesses two electrophilic α,β-unsaturated carbon groups that covalently bind to the in Keap1, and promote Nrf2/ARE binding to enhance antioxidant enzyme expression [34].Recently, curcumin was found to mediate the Nrf2/ARE pathway to protect TMJ chondrocytes [35], and BEAS-2B cells [36] from oxidative stress damage, resulting in decreased intracellular ROS levels, while promoting the transcriptional expression of Nrf2 along with its downstream antioxidant genes, including HO-1, NQO1. Zhou et al. [37] incubated human spermatozoa with the Nrf2 inhibitor Brusatol and curcumin, and found that curcumin inhibited ROS and MDA production by upregulating Nrf2 expression, thereby alleviating oxidative stress in spermatozoa. We speculate that curcumin enhances the antioxidant capacity of sperm by improving the nuclear translocation of Nrf2 in a way that regulates the Keap1-Nrf2/ARE signaling pathway, promoting the binding of Nrf2 to ARE, scavenging free radicals, collectively increase antioxidant enzyme activity, and inhibiting the production of ROS.

During IVF, sperm viability is positively correlated with fertilization rates [38]. IVF is affected by various parameters, including the fertilization environment, and sperm and oocyte quality. Sperm quality determines the outcome of IVF and subsequent embryonic development, and low-quality sperm reduces the embryo growth and development. It has been found that semen cryopreservation adversely affects sperm fertilization ability and embryo development, impacts which can be mitigated by the addition of antioxidants, such as reduced GSH [39], astragali polysaccharide [40], and melatonin [41]. In the current experiment, IVF experiments using bovine oocytes showed that the addition of 25 µM curcumin increased the fertilization and blastocyst formation rates from frozen-thawed spermatozoa. Research finding, reducing ROS levels in spermatozoa improves sperm viability and plasma membrane integrity, thereby enhancing fertility [42]. This is consistent with the results of the current experiment, suggesting that curcumin improves sperm antioxidant capacity by inhibiting ROS production, thereby improving fertilization with frozen–thawed sperm.

In vitro, the maturation of oocytes is an intricate process, wherein the formulation and conditions of the IVM medium, together with the intrinsic quality of the oocytes, are pivotal to maturity and development. Oocytes developed in IVM culture exhibit reduced quality and embryonic development relative to in vivo maturation [43]. When cultured in vitro, oocyte culture conditions are three to four times more oxygenated than they are in the reproductive tract, making oocytes more susceptible to free radicals and ROS accumulation [8]. Excessive ROS inhibit the antioxidant system of oocytes, leading to oxidative stress that negatively impacts reproductive physiology, including oocyte maturation, ovulation, and embryo development. Therefore, adding antioxidants to the IVM medium to protect oocytes from oxidative stress is a promising strategy.

It has been found that curcumin can alleviate oxidative stress, modulate the cell cycle, and reduce apoptosis to enhance oocyte maturation and blastocyst development [44]. It has also been applied in the prevention and treatment of reproductive disorders, including ovarian cancer [45,46], ovarian insufficiency [47], and polycystic ovary syndrome [48,49]. Hendarto et al. [50] demonstrated that curcumin promoted KitL protein expression in bovine oocytes, thereby regulating oocyte development. KitL, a growth factor produced by granulosa cells in ovarian follicles, binds to the oocyte receptor c-Kit to regulate both oocyte and follicle development. The current study examined the maturation and development of bovine oocytes by incorporating varying concentrations of curcumin to the IVM medium. Our results showed that curcumin significantly increased the maturation rate of bovine oocytes to the GVBD and MII stages and reduced the apoptosis rate. However, curcumin has been demonstrated to trigger apoptosis and developmental impairment in mouse blastocysts [51]. Chen et al. [52] showed that the incorporation of 20 µM curcumin into IVM medium impeded the maturation and fertilization rates of mouse oocytes during the MII stage. Contrary to the current results, curcumin-induced inhibition of oocyte maturation and development was mainly found in mice, whereas the oocyte maturation promotion effects of curcumin in livestock animals, such as pigs and cows, may reflect differences in animal species or dosage problems.

GSH as a cofactor of the GPX and glutathione reductase systems can facilitate the reduction of hydrogen peroxide, react with ROS, and regulate enzyme activity. In addition, GSH has been documented to significantly influence oocyte maturation, fertilization, and embryonic development in many species, including buffalo [53], pig [54], and sheep [55]. It was found that the reduction of GSH synthesis impeded the maturation of bovine oocytes to blastocyst stage, while supplementation of GSH in an in vitro medium had the opposite effect [56]. Therefore, GSH content in oocytes can be used to check the antioxidant capacity and also assess the maturity of oocytes. The present experiment showed that the addition of curcumin to oocytes IVM medium significantly increased GSH and MMP levels, and suppressed ROS and MDA, suggesting that curcumin was effective at improving the development of oocytes during IVM by improving the antioxidant capacity. Namula et al. [21] induced oxidative stress in porcine oocytes using H_2_O_2_ and found that curcumin supplementation to the maturation medium improved the maturation rate by enhancing antioxidant capacity and reducing DNA fragmentation, thereby promoting in vitro development of porcine oocytes. These findings further support the conclusion that curcumin protects oocytes from oxidative stress by enhancing antioxidant properties, ultimately improving the maturation and development of animal oocytes in vitro.

During IVF, fertilization, cleavage, and blastocyst rates are key indicators to evaluate the developmental efficiency of oocytes. In this study, the rate of fertilization and blastocyst formation was significantly increased in mature oocytes cultured with IVM medium containing 25 or 50 µM curcumin as compared to the control group. GSH is a crucial element in facilitating the depolymerization of sperm chromatin, reducing disulfide bonds in the sperm nucleus, and promoting the creation of male prokaryotic nuclei and early embryonic development during fertilization [57,58]. Moreover, research indicates a strong correlation between GSH concentrations in mouse oocytes and the rate of blastocyst formation [59]. These experiments demonstrate that curcumin supplementation increases GSH levels in oocytes, suggesting that curcumin may enhance oocyte fertilization rates and blastocyst formation by elevating GSH levels. Oocyte quality influences the effectiveness of IVF. Given that bovine oocytes are retrieved from the ovaries of slaughterhouses, the quality of oocytes in these studies may be affected by the different developmental status of individual animals and by factors such as transportation, which may lead to variations in the observed results.

## 5. Conclusions

To conclude, the current findings show that incorporating curcumin into both the bovine sperm cryopreservation solution and the oocyte IVM medium significantly improved the quality of frozen–thawed sperm, antioxidant activity, oocyte maturation, IVF rate, and embryonic development. The best results were observed with 25 µM curcumin, suggesting that curcumin could be a promising antioxidant for livestock reproduction procedures in vitro. It may help prevent oxidative stress during bull sperm cryopreservation, IVM, and embryonic development. These results indicate that curcumin holds potential as an effective antioxidant for enhancing livestock reproduction, with the potential to mitigate oxidative stress during bull sperm freezing, preservation, IVF, oocyte maturation, and embryonic development.

## Figures and Tables

**Table 1:** Effect of curcumin addition to in vitro maturation medium on meiotic capacity and DNA fragmented nuclei in bovine oocytes.

Concentration of Curcumin (µM)	No. of Examined Oocytes	No. (%) of Oocytes with		No. (%) of Oocytes with DNA Fragmented Nuclei
		GVBD	M II	
CT (0)	108	103 (95.4 ± 2.3)	57 (52.8 ± 1.8) ^c^	12 (12.0 ± 1.9) ^a^
**5**	105	101 (96.2 ± 2.1)	59 (56.2 ± 2.5) ^b,c^	8 (7.6 ± 2.1) ^a,b^
**10**	107	101 (94.4 ± 1.6)	65 (60.8 ± 2.2) ^b^	6 (5.6 ± 2.4) ^b^
**25**	109	100 (91.7 ± 1.9)	80 (73.4 ± 1.8) ^a^	6 (5.5 ± 1.6) ^b^
**50**	108	103 (95.4 ± 2.0)	72 (68.5 ± 2.4) ^a^	5 (4.6 ± 1.9) ^b^

CT; control, GVBD; germinal vesicle breakdown, MII; metaphase II. Data are expressed as mean ± standard deviation. A total of four replicated experiments were conducted and analyzed, with n = 25–30 per group. Values with different superscript letters in the same column are significantly different (*p* < 0.05). Identical letter notation signifies no significant difference (*p* > 0.05).

**Table 2:** Effect of curcumin addition to maturation culture on the fertilization and development of bovine oocytes.

Concentration of Curcumin (µM)	No. Oocytes (n_1_)	Fertilization Rate (%)	No. Oocytes (n_2_)	No. (%) of Embryos	
				Cleaved	Blastocyst
CT	102	62 (61.8 ± 1.8) ^b^	186	161 (86.3 ± 2.1)	44 (23.5 ± 2.7) ^b^
25	106	78 (73.6 ± 2.3) ^a^	178	155 (86.8 ± 2.6)	64 (35.9 ± 1.9) ^a^
50	103	78 (75.7 ± 1.5) ^a^	185	156 (84.4 ± 3.4)	59 (32.0 ± 1.7) ^a^

Data are expressed as mean ± standard deviation. A total of four replicated experiments were conducted and analyzed, with n_1_ = 20–30 per group, n_2_ = 40–50 per group. Values with different superscript letters in the same column are significantly different (*p* < 0.05). Identical letter notation signifies no significant difference (*p* > 0.05).

## Data Availability

Data are contained within the article.

## Abbreviations

The following abbreviations are used in this manuscript:

CAT	catalase
SOD	superoxide dismutase
MDA	malondialdehyde
MMP	mitochondrial membrane potential
COCs	cumulus oocyte complexes
ROS	reactive oxygen species
GSH-PX	glutathione peroxidase
